# AMPK agonist AICAR ameliorates portal hypertension and liver cirrhosis via NO pathway in the BDL rat model

**DOI:** 10.1007/s00109-019-01746-4

**Published:** 2019-02-05

**Authors:** Liangshuo Hu, Lin Su, Zhixia Dong, Yunhua Wu, Yi Lv, Jacob George, Jianhua Wang

**Affiliations:** 1 Department of Hepatobiliary Surgery, First Affiliated Hospital of Xi’an Jiaotong University, Xi’an, 710061 Shaanxi China; 2 Storr Liver Unit, Westmead Millennium Institute and Westmead Hospital, University of Sydney, Westmead, NSW 2145 Australia

**Keywords:** Portal hypertension, Liver cirrhosis, AMP-activated protein kinase, AICAR, Nitric oxide

## Abstract

**Abstract:**

Recent studies have indicated that the Adenosine 5‘-monophosphate (AMP)-activated protein kinase (AMPK) pathway is closely involved in liver fibrosis and other fibrotic diseases. However, whether targeting the AMPK pathway can rescue liver fibrosis and its complications, such as portal hypertension, is unknown. This study aimed to explore the therapeutic value of AICAR (5-aminoimidazole-4-carboxyamide ribonucleoside), an agonist of the AMPK pathway, on liver fibrosis and portal hypertension in bile duct ligation (BDL) rats. In vitro experiments showed that the gene expression levels of TGF-b, a-SMA, and collagen 1 in primary rat hepatic stellate cells (HSCs) were significantly decreased after AICAR treatment. The p-eNOS expression and nitric oxide (NO) production were increased by AICAR administration in sinusoidal endothelial cells (SECs). For in vivo animal studies, AICAR acutely decreased portal pressure in the BDL and CCL4 fibrotic rats, but not in the partial portal vein ligation (PVL) rats, without changes in systemic hemodynamics. It was also observed by using intravital fluorescence microscopy that AICAR led to sinusoidal vasodilation in situ experiment. We propose that the relevant mechanisms may be related to the activation of the AMPK/NO pathway in SECs and that this activation promoted NO production in the liver, thereby promoting hepatic sinusoid microcirculation and decreased intrahepatic resistance. The results were verified using the NO inhibitor L-NAME. Chronic AICAR treatment also showed profound beneficial effects on the BDL model rats. The hemodynamic condition was greatly improved, but the positive effect could be partially blocked by L-NAME. Moreover, AICAR also decreased hepatic fibrogenesis in the BDL rats.

**Key messages:**

- Acute and chronic use of AICAR could alleviate portal pressure without changing systemic hemodynamics.
- AICAR induced sinusoidal vasodilation by improving NO bioavailability and ameliorating endothelial dysfunction in vivo and in vitro.
- AICAR could alleviate liver cirrhosis in the BDL model rats.

**Electronic supplementary material:**

The online version of this article (10.1007/s00109-019-01746-4) contains supplementary material, which is available to authorized users.

## Introduction

Liver fibrosis is one of the leading causes of mortality worldwide regardless of recent therapeutic advances [1]. It is a wound-healing response that engages a range of cell types and mediators to encapsulate injury. In addition to deposition of extracellular matrix components, the pathogenesis of liver fibrosis is also associated with vascular remodeling. Portal hypertension (PHT) is one of the major complications of liver cirrhosis and is characterized by increased hepatic portal vein pressure gradient (HPVG) [2]. To date, upper gastrointestinal bleeding and hepatic failure caused by severe PHT still have high mortality rates in clinical settings [2, 3].

The pathophysiological process of PHT is complicated. Unbalanced vasoactive factors in the hepatic sinusoid and splanchnic circulatory system are considered the main contributors to the elevated portal pressure [4]. Among them, nitric oxide (NO) is one of the most important vasoactive factors in sinusoidal microcirculation [5]. Under the pathological conditions of liver cirrhosis and PHT, NO production decreases in the liver because of the dysfunction of hepatic sinusoidal endothelial cells (SECs). In contrast, nonspecific production of NO significantly increases in the splanchnic circulation [6]. Of note, activated hepatic stellate cells (HSCs) participate in processes such as remodeling and capillarization of hepatic sinusoids, leading to elevated intrahepatic vascular resistance [7].

In the liver, the AMPK pathway is involved in a series of biological processes, including protein synthesis, cell proliferation, and energy metabolism [8]. Activation of the AMPK pathway can increase the NO level in liver cells and promote the apoptosis of HSCs [9, 10]. However, no published data are available with regard to the anti-PHT role of AMPK agonists in liver diseases.

AICAR (5-aminoimidazole-4-carboxyamide ribonucleoside) is an agonist of the AMPK pathway. Previous studies showed that AICAR treatment reduced tubulointerstitial and interstitial fibrosis [11, 12]. In this study, we aimed to determine whether AICAR could ameliorate portal hypertension in an animal model. Its impact on liver fibrosis in BDL rats would also be investigated.

## Materials and methods

### Animals

Healthy male Sprague–Dawley (SD) rats (4-month-old, weighing 200–400 g) were randomly selected for the study. All of the animals were fed ad libitum with a commercial diet and had continuous access to fresh water. All rats were housed under conditions of constant ambient temperature (22 °C), humidity, and a 12-h light–dark cycle. This study was approved by the Western Sydney Area Health Service Animals Ethics Committee.

### Study design

#### In vitro study

Primary SD rat hepatic non-parenchymal cells were extracted and cultured in vitro to examine the effect of AICAR on HSCs and SECs. Gene and protein expression and NO concentration were quantitated after treatments with different concentrations of AICAR. The contractive ability of HSCs was also analyzed using the gel contraction assay.

#### In vivo study

This was classified into acute and chronic experiments.

#### Acute in vivo experiment

1. Thirty rats were randomly divided into 5 groups: control (1 mL saline), AICAR 10 mg (50 mg/kg), AICAR 20 mg (100 mg/kg), AICAR 40 mg (200 mg/kg), and L-NAME + AICAR (1 mg/kg L-NAME and then 200 mg/kg AICAR). Hemodynamic changes after acute agent administration (intravenously) were observed in 4-week BDL rats. All of the agents were diluted with 1 mL of saline and given by intravenous injections through the femoral vein catheter. Hemodynamic data were recorded in the following 60 min. The changes in hepatic microcirculation were observed under an intravital fluorescence microscope at the same time. At the end of experiments, three groups of animal models (control, AICAR 40 mg, and L-NAME + AICAR) were euthanized and liver/blood specimens were collected for Western blot analysis and biochemical assays.
2. In the second part of the acute in vivo experiment, 24 SD rats were randomly divided into 4 groups: sham, BDL model, CCL4 model, and partial portal vein ligation (PVL) model. After the full time of modeling, hemodynamic changes after acute agent administration were observed. Then, forty milligrams of AICAR were diluted with 1 mL of saline and given by intravenous injections through the femoral vein catheter. Hemodynamic data were recorded 60 min after injection.

#### Chronic in vivo experiment

1. Thirty rats were randomly divided into 3 groups: the sham, control, and AICAR groups. Rats in the sham group only separated the common bile duct adjacent tissues , and other rats received BDL modeling. Two weeks after the surgery, the rats in the AICAR group received daily subcutaneous injections with 200 mg/kg AICAR for the next 2 weeks, while the rats in the control and sham groups received the same volume of saline. The hemodynamic data were collected 4 weeks after the surgery. All of the rats were euthanized and livers/blood specimens were collected for the following experiment.
2. For the second part of the chronic in vivo experiment, 40 SD rats were randomly divided into 4 groups: control, L-NAME, AICAR, and AICAR + L-NAME. Two weeks after the surgery, the rats in the AICAR group were subcutaneously injected with 200 mg/kg per day of AICAR; those in the L-NAME group were orally administered 1 mg/kg per day of L-NAME and those in the AICAR + L-NAME group received both the agents, all for 2 weeks. Rats in the control group received the same volume of saline subcutaneous injection for 2 weeks. The next steps were the same as in the first phase of the chronic in vivo experiment.

Other materials and methods are described in supplementary information.

### Statistical analysis

All of the calculations were performed using SPSS 22.0 software. Quantitative data were expressed as the means ± standard deviations. Comparisons between two groups were analyzed using the Student’s *t* test. Two-way analysis of variance test was used for comparing more than two groups. A *P* value < 0.05 was considered statistically significant.

## Results

### AICAR treatment inhibits HSC contractility via activated the AMPK/NO pathway in vitro

Primary hepatic HSC and SEC cells were successfully extracted from SD rats and cultured. The TGF-β and α-SMA mRNA levels decreased to 56.4% and 64.6%, respectively, when the HSCs were treated with 1 mmol/L AICAR. mRNA levels were further decreased to 32.8% and 47.7%, respectively, after the cells were treated with 5 mmol/L AICAR (Supplementary Fig. 1A and 1B). The eNOS mRNA level did not change, while the iNOS mRNA level increased dramatically after the 1 mmol/L but not the 5 mmol/L AICAR treatment (Supplementary Fig. 1C and 1D). COL-1 decreased to 47.1% after 5 mmol/L AICAR treatment (Supplementary Fig. 1E). Because no significant difference was found between 1 and 5 mmol/L AICAR, the former was chosen for the following study. There was no difference between the control and AICAR-treated group in NO levels in the culture medium of HSCs (Supplementary Fig. 2A). To further verify the results, the protein expression was tested using Western blot analysis. The phosphorylated AMPK level increased to 173%, the α-SMA level decreased to 36.9%, and the iNOS level increased 2.26 times in the 1 mmol/L treated group compared with the control group (Supplementary Fig. 2B–F).

The primary SECs were treated with 1 mmol/L AICAR. The phosphorylated AMPK and eNOS protein levels increased to 154% and 201%, respectively, compared with the control group (Supplementary Fig. 3B–F). The NO concentration in the SECs culture medium increased 179% in the AICAR-treated groups (Supplementary Fig. 3A).

**Fig. 1:**
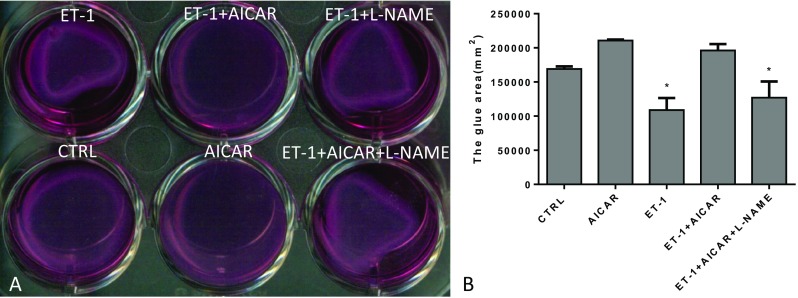
Gel contraction assay (*n* = 6, compared with the CTRL, *P < 0.05). **a** The gross evaluation using gel shrinkage test; **b** The area of gel shrinkage in different groups

In the gel contraction assay, the surface area of the gel became stable after 4 h of treatment with different agents. The gel treated with ET-1 contracted markedly, while the AICAR-treated gel had no obvious contraction compared with the control group. It suggested that AICAR could inhibit the ET-1 effect on the shrinkage of the gel. However, this effect could be partly suppressed by L-NAME (Fig. 1a, b).

### AICAR treatment decreases PVP in the acute in vivo experiment

**Fig. 2:**
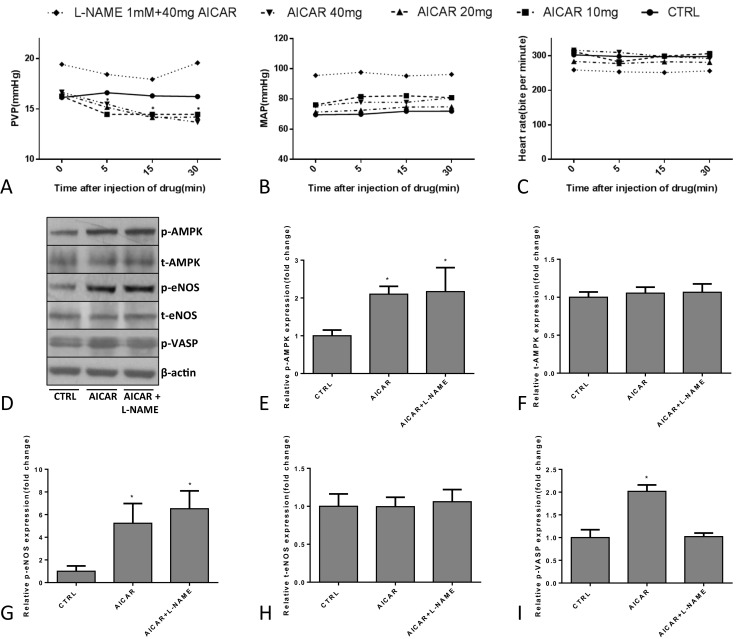
Hemodynamic detection in groups treated with different drugs in first part of the acute in vivo experiment in BDL ratsHJM901746(*n* = 6), **a**–**c** PVP, MAP, and heart rate change in groups treated with different drugs (*P < 0.05, compared with the CTRL); **d**–**i** Western blot analysis of the p/t-AMPK, p-eNOS/t-eNOS, and p-VASP protein expression in different groups (*P < 0.05, compared with the CTRL)

In the first part of the acute in vivo experiment with BDL rats, PVP decreased visibly after different doses of AICAR administration and remained stable for at least 60 min, especially in the 40 mg AICAR-treated group. However, this was blocked by the NO antagonist L-NAME (Fig. 2a). We also observed that AICAR injection had no influence on MAP and HR (Figs. 2b and 5c). AICAR and AICAR + L-NAME groups had higher expression of phosphorylated AMPK and phosphorylated eNOS than did the control groups. The expression of phosphorylated VASP was higher in the AICAR-treated groups compared with both control and AICAR + L-NAME groups (Fig. 2d–i).

**Fig. 3:**
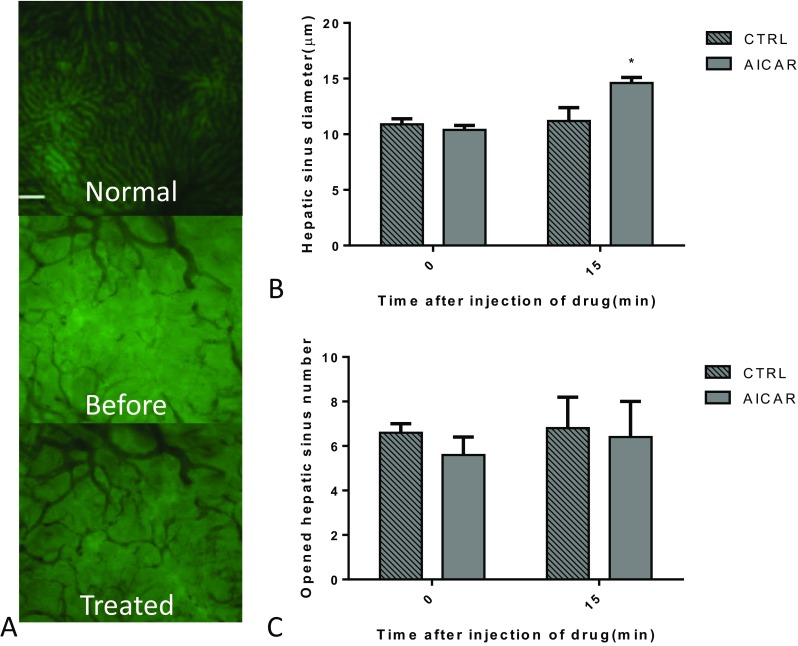
In vivo fluorescence indicated that acute AICAR treatment effectively promoted hepatic sinusoid microcirculation in BDL rats (*n* = 6, compared with the CTRL, *P < 0.05). **a** The gross evaluation of in vivo fluorescence when the cells were treated with AICAR. Normal: image of normal rat liver tissue; before: the BDL rat liver tissues before AICAR treatment; treated: BDL rat liver tissues after treatment with 40 mg AICAR for 15 min. **b** The hepatic sinus diameter changed after the cells were treated with 40 mg AICAR for 15 min. **c** The number of opened hepatic sinusoids after treatment with 40 mg AICAR for 15 min

Intravital fluorescence microscopy was used to examine how AICAR impacted intrahepatic vascular resistance in BDL rats. The changes became stable within 15 min after injecting the agents. The number and diameter of hepatic sinusoids before and 15 min after AICAR injection were tested (Fig. 3a). Hepatic sinus diameter dramatically increased after AICAR treatment compared with the control group (Fig. 3b) without changes in the number of opened hepatic sinusoids between the two groups (Fig. 3c).

In the second part of the acute in vivo experiment, three different PHT models were used to determine whether AICAR could effectively decrease PVP and its impact on the peripheral circulation. PVP was distinctly decreased after AICAR administration in the BDL and CCL4 groups, but not in the sham or PVL groups (Supplementary Fig. 4A). Additional data also showed that acute AICAR injection did not change MAP, HR, or the cardiac index in any of these four groups (Supplementary Fig. 4B–D).

### AICAR alleviates PHT and liver cirrhosis in BDL rats

**Fig. 4:**
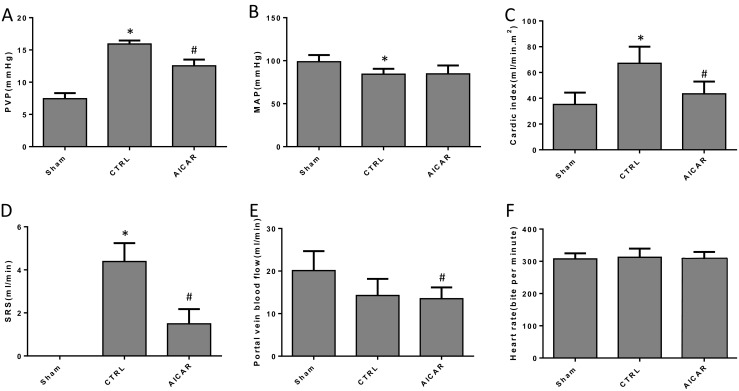
Hemodynamic detection in the first part of the chronic in vivo experiment (*n* = 6, compared with the Sham, *P < 0.05, compared with the CTRL, ^**#**^*P* < 0.05). **a**–**f** The changes of PVP, MAP, CI, SRS blood flow, portal vein blood flow, and HR in different groups

The hemodynamic changes were detected to investigate the long-term effects of AICAR in BDL rats. The PVP in the AICAR-treated group was much lower than in the BDL control group (Fig. 4a). MAPs in the AICAR-treated and BDL control groups were lower than in the sham group, but no difference was found between the first two groups (Fig. 4b). The cardiac index was lower in the sham and AICAR-treated groups than in the BDL control group (Fig. 4c). The blood flow of SRS could not be tested in the sham operation group and was higher in the BDL control group compared with the AICAR-treated group (Fig. 4d). The portal vein blood flow of the sham group was lower than in the other two groups and no difference was observed between the AICAR treated and control group (Fig. 4e). The HR in the three groups showed not difference (Fig. 4f).

**Fig. 5:**
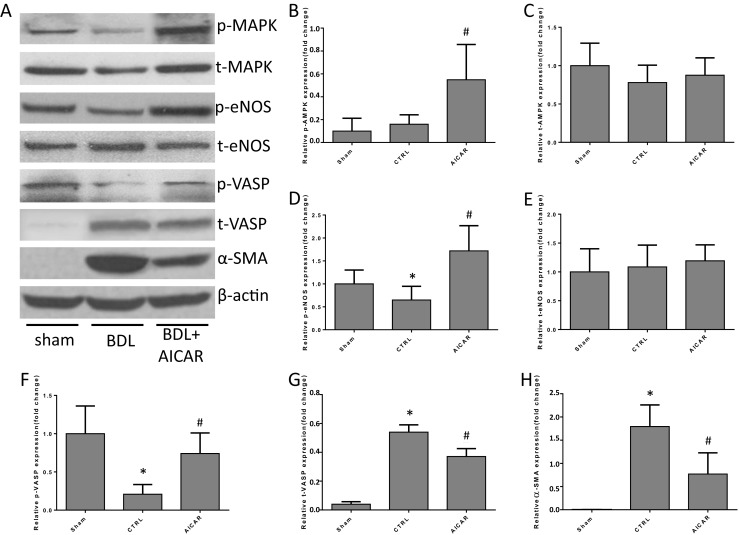
Protein expression of the liver tissue in the first phase of the chronic in vivo experiment (*n* = 6, compared with the Sham, *P < 0.05, compared with the CTRL, ^**#**^*P* < 0.05). **a**–**h** Western blot analysis of the p/t-AMPK, p/t-eNOS, p/t-VASP, and α-SMA protein expression in different groups

Relative indicators were detected to determine whether the long-term use of AICAR could alleviate liver cirrhosis in the BDL model. The mRNA levels of TGF-β, α-SMA, COL-1, TIMP-1, and CTGF were significantly lower in the AICAR-treated group than in the BDL control group (Supplementary Fig. 5A–E). The mRNA levels of iNOS and eNOS were higher in the BDL control and AICAR-treated group than in the sham operation group. However, no difference was found between the latter two groups (Supplementary Fig. 5F and 5G). The protein expression of p/t-AMPK, p/t-eNOS, p/t-VASP, and α-SMA in the liver tissue of different groups was assessed using Western blot analysis to further verify the findings. Protein levels of phosphorylated AMPK and eNOS were significantly higher in the AICAR-treated group than in the BDL control and sham operation group. Phosphorylated VASP was higher in the liver tissue of the AICAR treated group than the BDL control group. Total VASP and α-SMA were lower in the AICAR group than the BDL control group (Fig. 5).

**Fig. 6:**
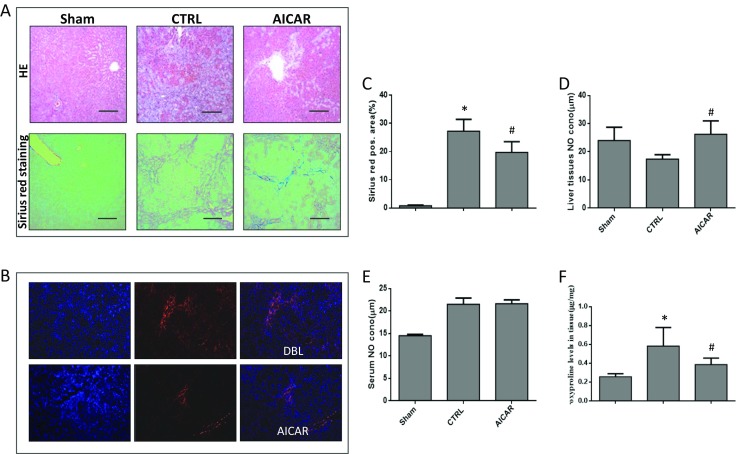
Fibrosis was reduced by AICAR treatment in BDL rats (*n* = 6, compared with the Sham, *P < 0.05, compared with the CTRL, ^**#**^*P* < 0.05). **a** H&E and Sirius red staining of liver tissues in different groups (original magnification × 100); **b** the immunohistofluorescence staining (α-SMA) of liver tissues in different groups (original magnification × 100); **c** the representative Sirius red staining of liver tissues in different groups; **d** the concentration of NO in liver tissues; **e** concentration of NO in serum of peripheral blood; **f** hydroxyproline level in liver tissues

The H&E and Sirius red staining of liver tissue in different groups showed that fibrosis was reduced by AICAR treatment (Fig. 6a, c), which was also verified by immunofluorescence staining of α-SMA (Fig. 6b). The hydroxyproline level dramatically decreased in the liver tissues of the AICAR-treated groups compared with the BDL control group (Fig. 6f). The concentration of NO in the liver tissue was higher in the AICAR-treated group than in the BDL control group (Fig. 6d). However, there was no difference in the NO level in the serum from peripheral blood between the two groups (Fig. 6e).

The aspartate aminotransferase (AST), alanine aminotransferase (ALT), total bilirubin, albumin, total protein, and gamma-glutamyl transferase (GGT) levels in the serum were evaluated to investigate the effect of AICAR on liver function (Supplementary Fig. 6). The total bilirubin dramatically decreased after AICAR treatment. ALT and AST levels in the control and AICAR groups were significantly higher than in the sham operation group, but no difference was observed between the former two groups. The albumin levels in the sham and AICAR groups were distinctly higher than those in the control group. However, the total serum protein level showed no difference among the three groups. The GGT levels in the BDL control and AICAR-treated groups found no difference as well.

### L-NAME blocks the effects of AICAR on PHT in BDL rats

**Fig. 7:**
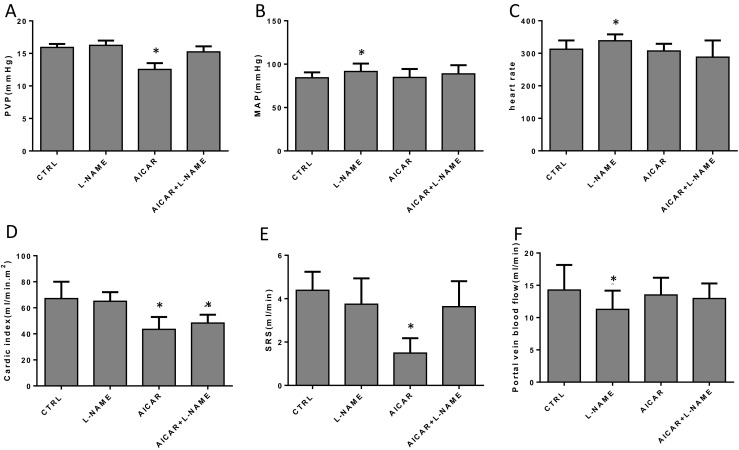
Hemodynamic detection in different groups in the second phase of the chronic in vivo experiment (*n* = 6, compared with the CTRL, *P < 0.05). **a**–**f** The changes in PVP, MAP, HR, CI, SRS, and portal vein blood flow in different groups

The second part of the chronic in vivo experiment was performed to find out whether AICAR decreased portal pressure via the NO pathway by treating the BDL model rats with both AICAR and the NO inhibitor L-NAME. Rats in the L-NAME group had increased MAP and decreased portal vein and SRS blood flow compared with the BDL control group. No difference in PVP or CI was found between these two groups, (Fig. 7b, c). The PVP and SRS blood flow were significantly increased in the AICAR + L-NAME group compared with the AICAR groups. No difference in MAP, HR, CI, or portal vein blood flow was found between them (Fig. 7d). The SRS blood flow in the AICAR-treated group was the lowest among these four groups and the effect could be blocked by L-NAME administration (Fig. 7a, e).

**Fig. 8:**
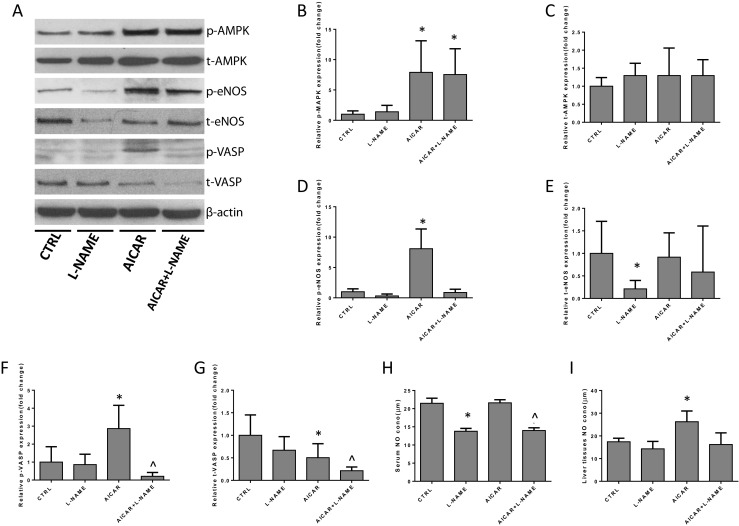
Mechanism underlying the inhibitory effect of AICAR on the change in portal hypertension in the in vivo experiment (*n* = 6, compared with the CTRL, *P < 0.05, compared with AICAR group, ^**^**^*P* < 0.05). **a**–**g** The Western blot analysis of p/t-AMPK, p/t-eNOS, and p/t-VASP protein expression in the liver tissue of different groups. **h** The concentration of NO in serum of peripheral blood; **i** the concentration of NO in liver tissues

Western blot analysis showed that L-NAME did not affect AMPK phosphorylation or expression. However, it was found to block NO/VASP activities. L-NAME also decreased the protein expression of eNOS (Fig. 8a–g). NO concentration in the liver tissue and plasma also decreased in the AICAR + L-NAME group when compared with the AICAR group (Fig. 8h, i).

## Discussion

Portal hypertension is a common and severe clinical consequence of chronic liver disease associated with significant morbidity and mortality [13, 14]. The non-parenchymal cells play critical roles in liver diseases and intrahepatic vascular remodeling [15, 16]. Contractility of activated HSCs contributes to the reversible increased intrahepatic resistance. The vasoactive factors are unbalanced in the hepatic sinusoid which is related to dysfunction of hepatic sinusoid endothelial cells. The ideal treatment for PHT is proposed to specifically target hepatic vasoactive factors such as NO but exert no actions on the systemic circulation [17]. Numerous efforts to modulate or interrupt intrahepatic resistance have been investigated by specifically targeting the non-parenchymal cells especially HSCs with AT1R blockers, Rho-kinase inhibitors, or interferon-gamma [18–22]. Approaches aimed on the intrahepatic vasoactive factors have also been tested in animal models. Among them, NO-related agents hold great potential, including NO delivery [23, 24], NO synthase modulators [25], and NO enhancers such as statins and obeticholic acid [26–29]. Clinical studies appeared that statins, which mainly act on Rho GTPases and Kruppel-like transcription factor 2 (KLF2) signaling, increased the survival of cirrhotic patients with portal hypertension, especially those with concomitant cardiovascular diseases [30–32]. For splanchnic circulatory, non-selective β-blockers reduce portal pressure by lowering portal tributary blood flow and have been widely studied for their effect on preventing variceal bleeding [33, 34]. Carvedilol, a non-selectiveβ-blocker with additional anti-α1-adrenergic activity, further decreases HVPG better than propranolol and could possibly replace propranolol as primary prophylaxis of variceal bleeding in cirrhotic patients [35]. However, there is still no FDA approved specific anti-PHT medicine in the clinical setting.

The present study demonstrated that AICAR, an agonist of the AMPK pathway, reduces PVP in rats with established portal hypertension and liver cirrhosis. The data showed that AICAR also ameliorated liver cirrhosis in the BDL rat model. We next found that AICAR improved intrahepatic vascular tone without adverse effects of systemic circulation. In acute AICAR injection experiment, hepatic sinusoid diameter was enlarged and intrahepatic resistance decreased and this could be observed by intravital fluorescence microscopy.

Our previous studies proved that adiponectin activates the AMPK/iNOS pathway in HSC and induces a highly elaborate NO production, which partly explained the anti-fibrotic actions of adiponectin. In this study, AICAR—a classical AMPK agonist, also induces iNOS expression/NO production and supported our concept, although there was some controversy [36, 37]. In addition, AICAR is reported to mainly activate the AMPK/eNOS pathway in SECs [38, 39]. We next performed SEC in vitro experiments and observed a remarkable augmented effect of p-eNOS/NO production on SECs by AICAR. To confirm NO’s actions by AICAR, we further examined NO inhibition (L-NAME) assay in cultured HSC-gel contraction in vitro experiment. Additional data confirmed that AICAR-induced NO alleviated HSC contractility. Based on these data, we are confident that AICAR is able to improve the intrahepatic vascular resistance, at least through increased SEC/HSC NO production. Since phosphorylated VASP is a sensitive monitor of nitric oxide/cGMP signaling and endothelial function [40], we used rat liver samples to analyze p-VASP expression. Interestingly, p-VASP was significantly activated by AICAR and this effect could be blocked by L-NAME.

We observed that acute AICAR administration resulted in PVP reduction without deleterious effects on systemic circulation in two animal models (BDL and CCl4). We further assessed BDL rat liver fibrosis development and noticed an anti-fibrotic effect of AICAR. Since the PVL model does not involve pathological changes in the liver, we observed no change of PVP and systemic blood pressure, further indicating that AICAR could improve NO deficiency in cirrhotic liver and rescue portal hypertension. Chronic AICAR treatment could also alleviate PHT and had a good impact on systemic and splanchnic circulation. We believe that the amelioration of fibrosis is the factor other than AMPK/NO signaling that is involved in the decreased intrahepatic vascular resistance and improvement in splanchnic circulation for chronic AICAR administration. In addition, reduced α-SMA expression in HSCs was indicated by immunofluorescence of liver tissues. This may also contribute to reduced contractibility of the hepatic sinusoid and intrahepatic resistance. We found the NO concentration to be significantly increased only in the liver, and not in the peripheral circulation. This may be one reason why the chronic effect of AICAR was not obvious in the peripheral circulation. Why AICAR did not cause meaningful reduction of systemic pressure is still sophisticated to explain. Previous studies reported that AMPK activation reduced blood pressure in obese [41] and hypertensive rodents [42, 43] but had no impact of blood pressure in normal animals [44, 45]. AICAR largely promoted nitric oxide synthesis in microvascular perfusion rather than macrovascular perfusion [46]. In the cirrhotic model, the nonspecific increase of eNOS expression in the aortic and splanchnic endothelial cells could also block the effect of AICAR by the negative feedback mechanism [47]. Our data are also similar to a recent report by Schwabl P and he found riociguat, a soluble guanylate cyclase (sGC) stimulator, reduced portal pressure in cirrhotic rats without an impact on systemic effect [48]. sGC is the main target of NO downstream signaling that mediates vasodilation by catalyzing the reaction from GTP to cGMP [6]. Schwabl observed that the sGC enzyme was highly expressed in the liver but not in the systemic circulation. Thus, NO-induced sGC stimulation could exert its vasodilatory effects only in the intrahepatic microcirculation in the cirrhotic model but not the PVL model. Further mechanisms still need to be ascertained.

In this study, the anti-fibrotic action of AICAR was also noted. AICAR significantly decreased hepatic hydroxyproline content and improved the pathological change of BDL rats. The expression of TGF-β, α-SMA, TIMP, and CTGF (the indicators of hepatic fibrosis) was decreased in the chronic AICAR-treated BDL rats. Previous studies have shown that ACIAR alleviated carbon tetrachloride-induced acute hepatitis in mice [37]. This may add an alternative explanation regaiding how AICAR reduces portal pressure in our BDL rats. One reason is increased intrahepatic NO production, and another reason could be decreased liver fibrosis and improved liver structure. It is also not clear, in terms of the mechanism, how AICAR ameliorate liver fibrosis. Chen et al. once reported that melatonin could protect the liver from BDL-induced mitochondrial oxidative stress via the AMPK-SIRT3-SOD2 pathway [49]. Yang et al. found that ursolic acid offers protective effects on BDL-induced liver injury in mice via LKB1/AMPK signaling [50]. In terms of our study, increased NO production (particularly derived from SECs) by AICAR may also largely improve subsequent liver fibrosis in BDL rats. We also found that AICAR could improve the inflammation processes in BDL rats though the AMPK/non-canonical NF-κB pathway, but the underlying mechanism needs further research.

The present study has several limitations. First, our research mainly focused on the mechanisms of how AICAR works in the improvement in the hepatic vascular tone or hepatic microvascular dysfunction. Data from our research showed that no systemic effects were observed for AICAR administration, but more insight is still needed. Second, this study found the effect of AICAR on liver cirrhosis only in the BDL model. These findings need to be confirmed in other models of liver cirrhosis and the underlying mechanism needs further research. Whether L-NAME could inhibit the effect of AICAR on liver cirrhosis is unknown and needs further investigation.

## Conclusions

AICAR treatment improves PHT and hepatic fibrogenesis on cirrhotic rat model. The mechanism is supposed to be related to the activation of the AMPK/NO pathway in SECs, which promotes hepatic sinusoidal microcirculation and decreases intrahepatic resistance.

## Electronic supplementary material

ESM 1 (DOC 51 kb)

ESM 2 (PPTX 379 kb)
