# Supplementary material for: AMPK agonist AICAR ameliorates portal hypertension and liver cirrhosis via NO pathway in the BDL rat model
Source: J Mol Med (Berl). 2019 Feb 5;97(3):423–34. doi: 10.1007/s00109-019-01746-4 (PMC6394556; doi:10.1007/s00109-019-01746-4)
Supplement: Supplementary file 1 — (DOC 51 kb) [file 109_2019_1746_MOESM1_ESM.doc]

**Supplemental materials and methods**

Isolation and culture of liver nonparenchymal cells.

HSCs of SD rats were obtained by two-step (collagenase B and pronase E) perfusion methods, and SECs were further obtained and purified by elutriation as previously described. HSCs and SECs were separately cultured for 7 days and 2 days, respectively. They were then treated with 1 µmol and 5 µmol doses of AICAR, respectively, for 1 h, while the control group was treated with the same volume of PBS.

HSC viability was assessed by trypan blue exclusion and was routinely over 95%. Purity was at least 95% as determined by morphology, vitamin A autofluorescence and desmin positivity.

The viability of SECs was > 98% and purity at least 94% as determined by morphology (cobblestone appearance) and absence of latex bead phagocytosis.

Gel contraction assay

The gel contraction assay was used to test the contractility of HSCs. First, hydration protein glue plate with type I rat tail collagen solution was made. HSCs and SECs were collected and a mixed cell suspension (2.5 × 10^5^/mL) was made. The suspension was then added to the prepared hydration protein glue plate (2 mL for each hole). After cultivation overnight, the medium was changed to Dulbecco’s Modified Eagle Media (DMEM) with no fetal calf serum (FCS) and kept for 4 h. Then, 1 mM AICAR, 10 nM endothelin-1 (ET-1), 1 mM AICAR (preliminary treated for 1 h) + 10nM ET-1, 1 mM AICAR + 5 mM L-nitro-arginine methylester (L-NAME) (both preliminary treated for 1 h) + ET-1, 5 mM L-NAME (preliminary treated for 1 h) + ET-1, and same volume of PBS were added. In the next 6 h, the glue plate was photographed every hour, and its surface area in different groups was compared.

Animal model

BDL model

Full surgical procedures were performed under isoflurane (Ilium, Smithfield, Australia) anesthesia (2% isoflurane with 0.4 L/min O_2_ flow). Briefly, the common bile duct was double ligated and resected at the midpoint between two ligatures. Then, all of the rats were fed a normal diet for 4 weeks.

CCL4 model

Liver fibrosis and PHT were induced by CCL4 injection (intraperitoneally, 500 μL/kg in a 1:1 solution of olive oil, twice weekly for 8 weeks).

Partial portal vein ligation (PVL) model

Full surgical procedures were performed under anesthesia as described above. Briefly, the portal vein proximal to the bifurcation was exposed. A 3-0 silk ligature was made around the portal vein around a piece of PE-50 tubing (Clay Adams, Parsippany, NJ, US). The PE tubing was then removed and the abdomen closed. Then, all of the rats were fed a normal diet for 2 weeks.

All of the rats were maintained on a standard rat pellet diet and water ad libitum.

Hemodynamic measurements

For hemodynamic evaluation, the mean arterial pressure (MAP) and heart rate (HR), portal venous pressure (PVP), portal vein blood flow, splenorenal shunt (SRS) blood flow, and cardiac output were determined. After anesthesia, MAP and HR were measured using a pressure transducer (PowerLab Data Acquisition Systems, ADInstruments, Sydney, Australia) connected to the right femoral arterial catheter. PVP was measured with a pressure transducer connected to a polyethylene catheter (0.5 mm diameter), which was inserted into a small iliac vein and gently advanced up to the bifurcation of the superior mesenteric and splenic veins. The portal vein blood flow and splenorenal shunt (SRS) blood flow were measured using a transit-time ultrasound technique (T402-PP Two Channel PerivascularFlowmete, ADInstruments, Sydney, Australia). Cardiac output was measured using the dilution method (MLT1402 T-type Ultra Fast Thermocouple, ADInstruments, Sydney, Australia). The body temperature was maintained at 37°C for all of the surgical procedures.

Intravital fluorescence microscopy

The rats were injected with 0.2 mL of saline with green sodium fluorescein (1.5 µg/g, Sigma–Aldrich) through the portal vein, and the livers were observed under the fluorescence microscope. The hepatic sinus was assessed as follows. A six-plane element (200 × 200 µm^2^) was randomly picked during the microscopic examination, and the diameter and number of hepatic sinusoids were recorded.

Western blot analysis

The total proteins of tissues and cells were extracted using RIPA lysis buffer. Then, the proteins were resolved on 10% sodium dodecyl sulfate–polyacrylamide gels and transferred on to PVDF membranes. The membranes were blocked with 5% skim milk and incubated with primary antibodies overnight at 4°C, which included an anti-phospho-AMPK antibody (Thr172) (Cell Signaling, MA, US; 1:1000 dilution), anti-total AMPK antibody (Cell Signaling; 1:1000 dilution), anti-phospho-eNOS antibody (Cell Signaling; 1:1000 dilution), anti-total eNOS antibody (Cell Signaling; 1:1000 dilution), anti-phospho-vasodilator-stimulated phosphoprotein (VASP) antibody (Cell Signaling; 1:1000 dilution), anti-total VASP antibody (Cell Signaling; 1:1000 dilution), anti-α-smooth muscle actin (SMA) antibody (Cell Signaling; 1:1000 dilution), anti-β-actin antibody (Cell Signaling; 1:1000 dilution), anti-glyceraldehyde-3-phosphate dehydrogenase (GAPDH) antibody (Cell Signaling; 1:1000 dilution), and anti-total induced nitric oxide synthase (iNOS) antibody (Santa Cruz, MA, US; 1:1000 dilution). Then, the membranes were washed and incubated with secondary antibodies at room temperature for 2 h. Protein expression was detected using a chemiluminescence system (Millipore, US) according to the manufacturer’s protocol.

Real-time quantitative polymerase chain reaction

The real-time reverse transcription–polymerase chain reaction (RT-PCR) was performed to determine the messenger RNA (mRNA) levels of tumor necrosis factor alpha, transforming growth factor beta (TGF-β), iNOS, eNOS, α-SMA, tissue inhibitors of matrix metalloproteinase 1 (TIMP-1), connective tissue growth factor (CTGF), and collagen I. The RNA was first extracted using the RNA kit following the manufacturer’s protocol (UltraClean Tissue & Cells RNA Isolation Kit for cells and Qiagen RNeasy Kit for tissues). Then, reverse transcription was performed using a PrimeScript RT reagent Kit (Takara, Dalian, China). The real-time experiments were conducted on a polymerase chain reaction real-Time PCR detection system (ABI Biosystems, Hercules, Australia) using a SYBR Green real-time PCR master mix (Takara). The PCR conditions were as follows: 95°C for 2 min followed by 40 cycles of 95°C for 15 s, 60°C for 45 s, and 72°C for 30 s. The PCR primer sequences are shown in Supplementary Table 1. The comparative C(T) method was used to quantitate the expression of each target gene using GAPDH as the normalization control, and the result was calculated using the 2^-ΔΔCT^ method.

Nitrate/nitrite fluorometric assay

The NO level was tested using the nitrate/nitrite fluorometric assay kit (Sapphire, Australia) according to the manufacturer’s protocol. The NO2-/NO3-assay was undertaken on cell culture medium, serum, and liver homogenates collected from experimental cells and rats.

Hydroxyproline determination

Hydroxyproline content was determined using the hydroxyproline assay kit (Sigma–Aldrich) following the manufacturer’s protocol.

Histomorphological examination

Hematoxylin and eosin staining

After 24 h of fixation, the tissues embedded in paraffin were cut into 4 μm-thick serial paraffin sections. Some paraffin sections were stained with hematoxylin and eosin (H&E) to allow for visualization of the morphology and adhesion condition of the tissues.

Immunofluorescence

The fresh tissues were first embedded in OCT, kept at –80°C overnight, and then cut into 4 μm-thick serial paraffin sections. After fixing in 4% formalin for 30 min, the sections were washed and blocked using DAKO for 40 min, followed by incubation with α-SMA (1:500) at 4°C overnight. After two rounds of washing, the tissues were incubated with DAPI (Cell Signaling; 1:1000). Then, the sections were dehydrated, mounted, sealed, and kept in a dark environment

Sirius red staining

Collagen was stained using 0.1% picrosirius red (Direct Red 80; Sigma–Aldrich) and counterstained with Weigert’s hematoxylin. Japan). The percentage of the positively stained area was evaluated using ImagePro Plus 5.0 software (Leica Qwin.Plus, Leica Microsystem Imaging Solutions Ltd, Cambridge, UK) in eight randomly selected ﬁelds, and the average of the eight values was taken as the collagen content in the adhesions.

All of the histopathological evaluations of fibrosis and inflammation were viewed and evaluated by two pathologists who were blind to experimental groups from the Pathology Department of First Affiliated Hospital of Xi’an Jiaotong University. At least three randomly selected high-power fields were reviewed for each pathological section, and at least two sections for each rat were confirmed and scored.

**Supplement Figure Captions**

Supplemental Figure 1. Relative mRNA expression of TGF-β1, α-SMA, e-NOS, i-NOS, and COL-1 of HSCs when treated with AICAR (n = 3, compared with the CTRL, **P*<0.05). A-E: The relative expression of TGF-β1, α-SMA, e-NOS, i-NOS, and COL-1 in different groups.

Supplemental Figure 2. AICAR treatment could activate AMPK/NO pathway in primary HSC cells (n = 3, compared with the CTRL, **P*<0.05). A: The concentration of NO in HSC cell culture medium; B-F: the protein expression of iNOS, α-SMA, and p/t-AMPK in different groups.

Supplemental Figure 3. Effect of AICAR on primary hepatic SEC cells (n = 3, compared with the CTRL, **P*<0.05). A: The concentration of NO in SEC cell culture medium; B: The protein expression of p/t-AMPK and p/t-eNOS in AICAR-treated and control SEC cells; C-F: the protein expression of p-AMPK, t-AMPK, p-eNOS, and t-eNOS in different groups.

Supplemental Figure 4. Hemodynamic detection in second part of the acute *in vivo* experiment (each group, n = 6), A-D: PVP, MAP, heart rate and cardiac index change in animal models before and 60 min after treatment with AICAR (**P*<0.05, compared to pre-injection levels).

Supplemental Figure 5. Relative mRNA expression of liver tissues in the first phase chronic *in vivo* experiment (n = 6, compared with the Sham, **P*<0.05; compared with the CTRL, **^#^***P* <0.05). A–G: The relative expression of TGF-β1, α-SMA, COL-1, TIMP-1, CTGF, i-NOS, and e-NOS in different groups.

Supplemental Figure 6. Liver function test of different groups in the first phase chronic *in vivo* experiment (n = 6, compared with the Sham, **P*<0.05; compared with the CTRL, **^#^***P* <0.05). A-F: Liver function test results of total bilirubin, ALT, AST, albumin, total protein and GGT in different groups.

**Reference**

1. Dong Z, Su L, Esmaili S, Iseli TJ, Ramezani-Moghadam M, Hu L, Xu A, George J, Wang J (2015) Adiponectin attenuates liver fibrosis by inducing nitric oxide production of hepatic stellate cells. Journal of molecular medicine 93 (12):1327-1339. doi:10.1007/s00109-015-1313-z

2. Fizanne L, Regenet N, Wang J, Oberti F, Moal F, Roux J, Gallois Y, Michalak S, Cales P (2008) Hemodynamic effects of the early and long-term administration of propranolol in rats with intrahepatic portal hypertension. Hepatology international 2 (4):457-464. doi:10.1007/s12072-008-9070-5
