# Supplementary material for: AMPK agonist AICAR ameliorates portal hypertension and liver cirrhosis via NO pathway in the BDL rat model
Source: J Mol Med (Berl). 2019 Feb 5;97(3):423–34. doi: 10.1007/s00109-019-01746-4 (PMC6394556; doi:10.1007/s00109-019-01746-4)
Supplement: Supplementary file 2 — (PPTX 379 kb) [file 109_2019_1746_MOESM2_ESM.pptx]

## Slide 1
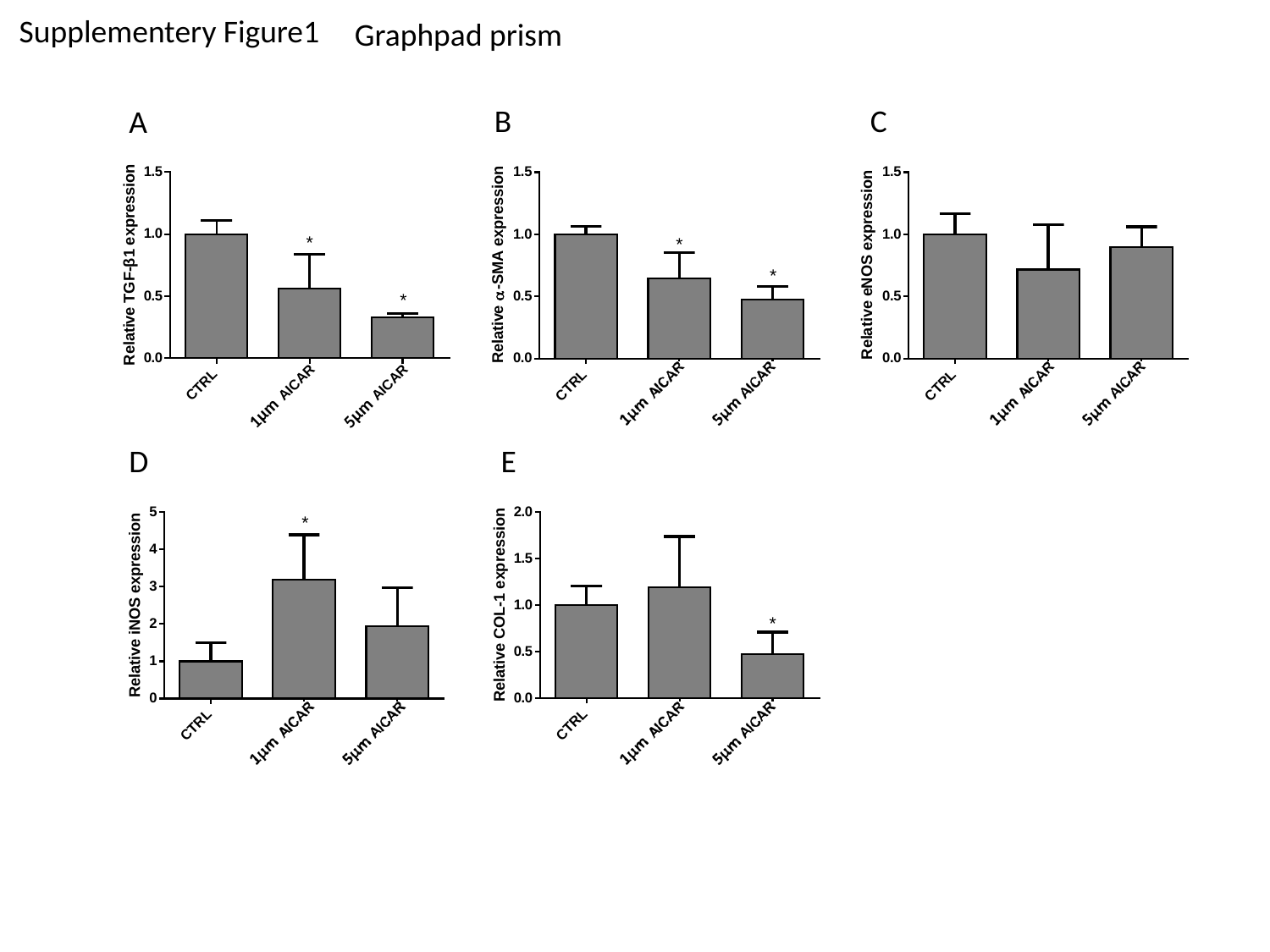

Supplementery Figure1
Graphpad prism
B
C
A
E
D
1μm
5μm

## Slide 2
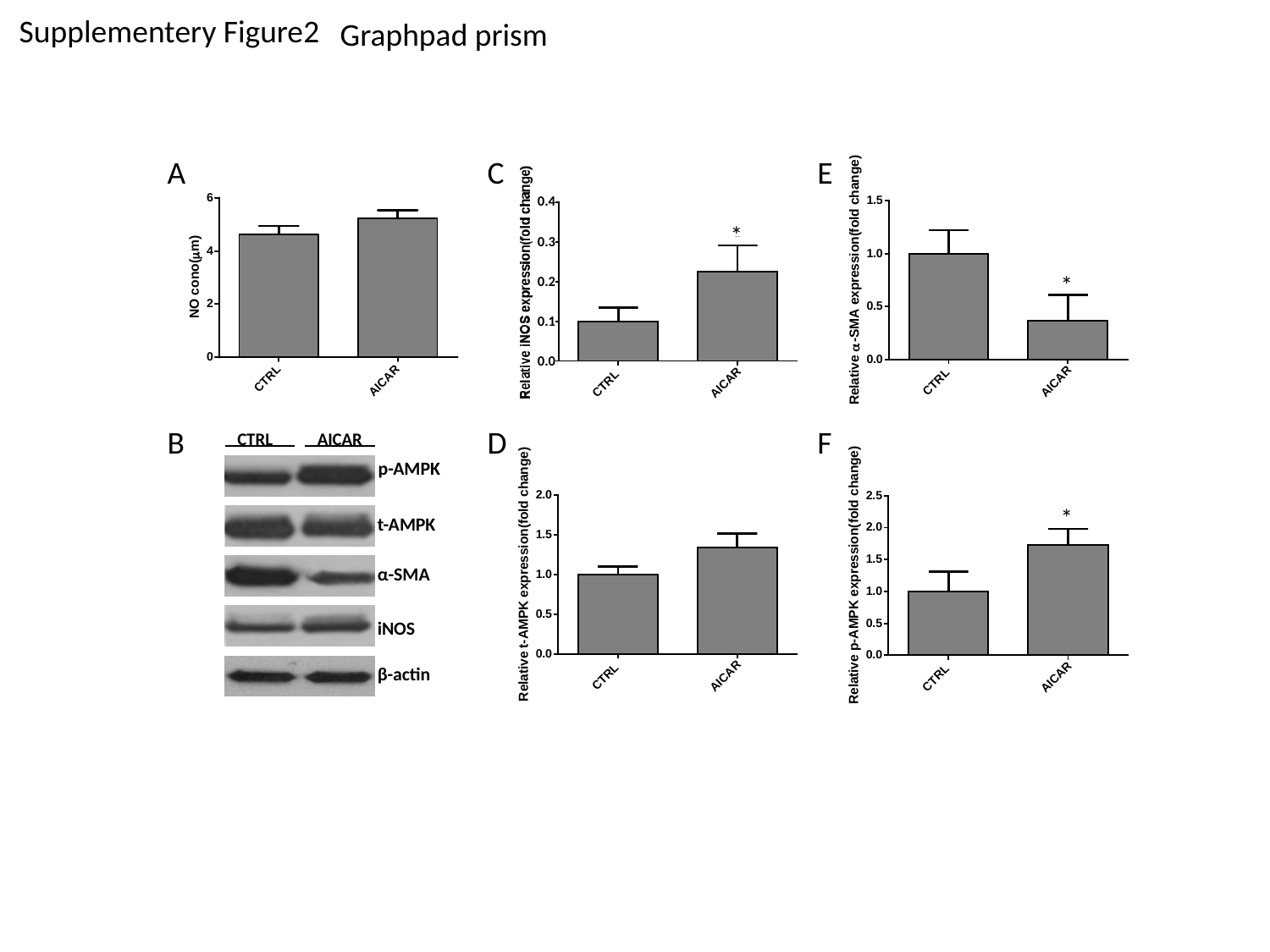

Supplementery Figure2
Graphpad prism
0.4
 *
0.3
 *
0.2
0.1
0.0
CTRL
AICAR
p-AMPK
 *
t-AMPK
α-SMA
iNOS
β-actin
A
C
E
B
D
F

## Slide 3
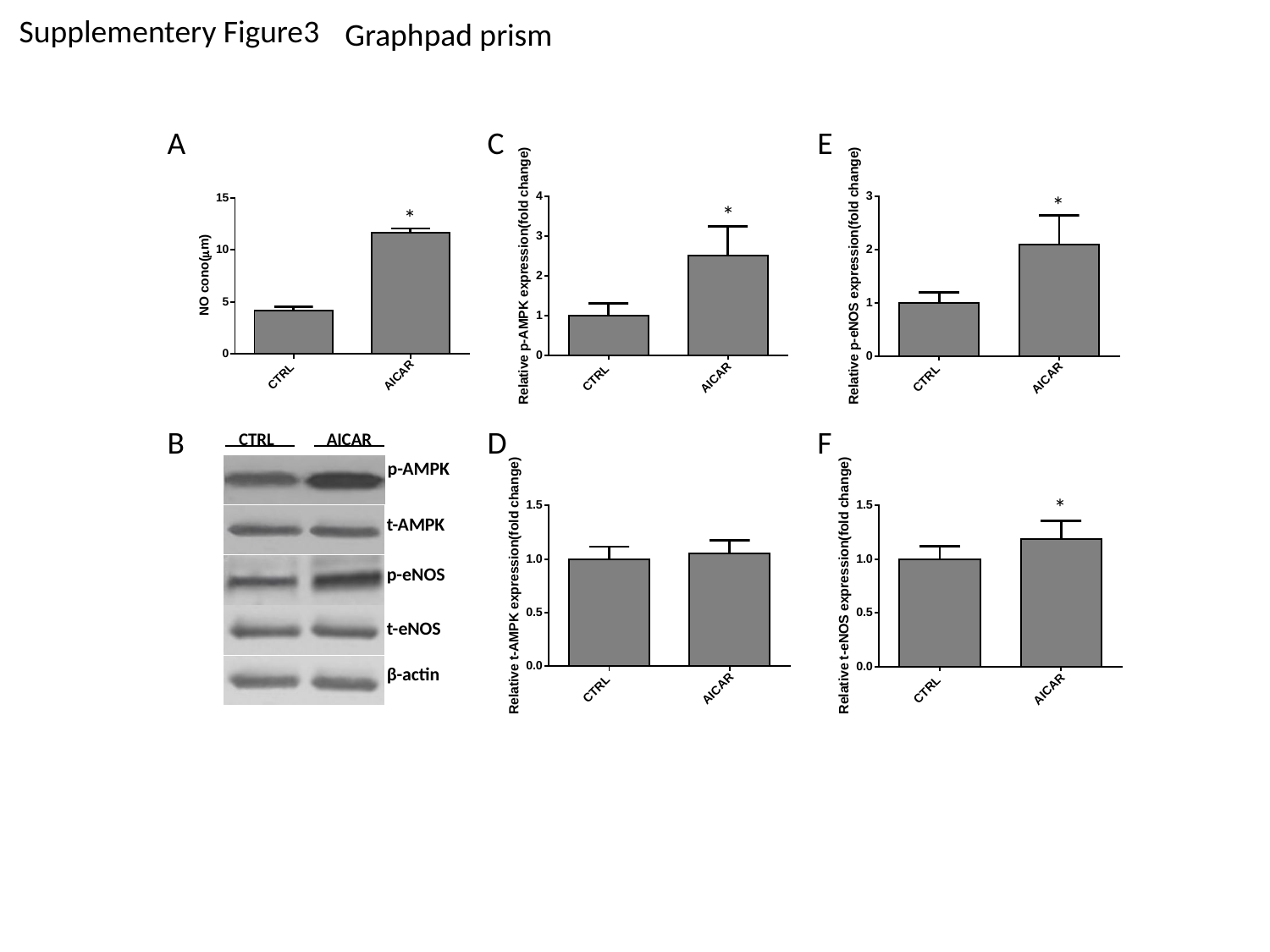

Supplementery Figure3
Graphpad prism
A
C
E
 *
 *
 *
CTRL
AICAR
p-AMPK
 *
t-AMPK
p-eNOS
t-eNOS
β-actin
B
D
F

## Slide 4
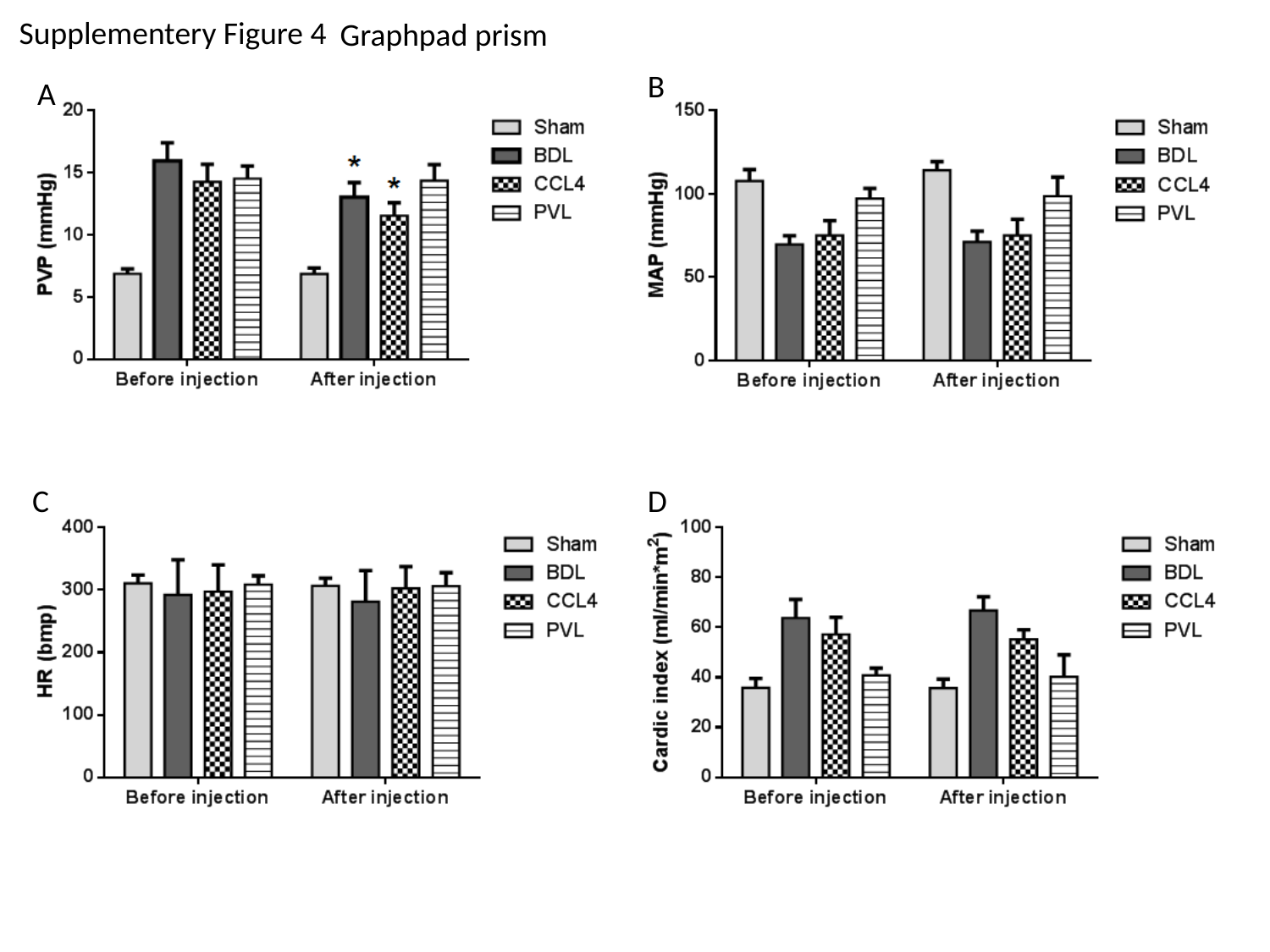

Supplementery Figure 4
Graphpad prism
B
A
C
D

## Slide 5
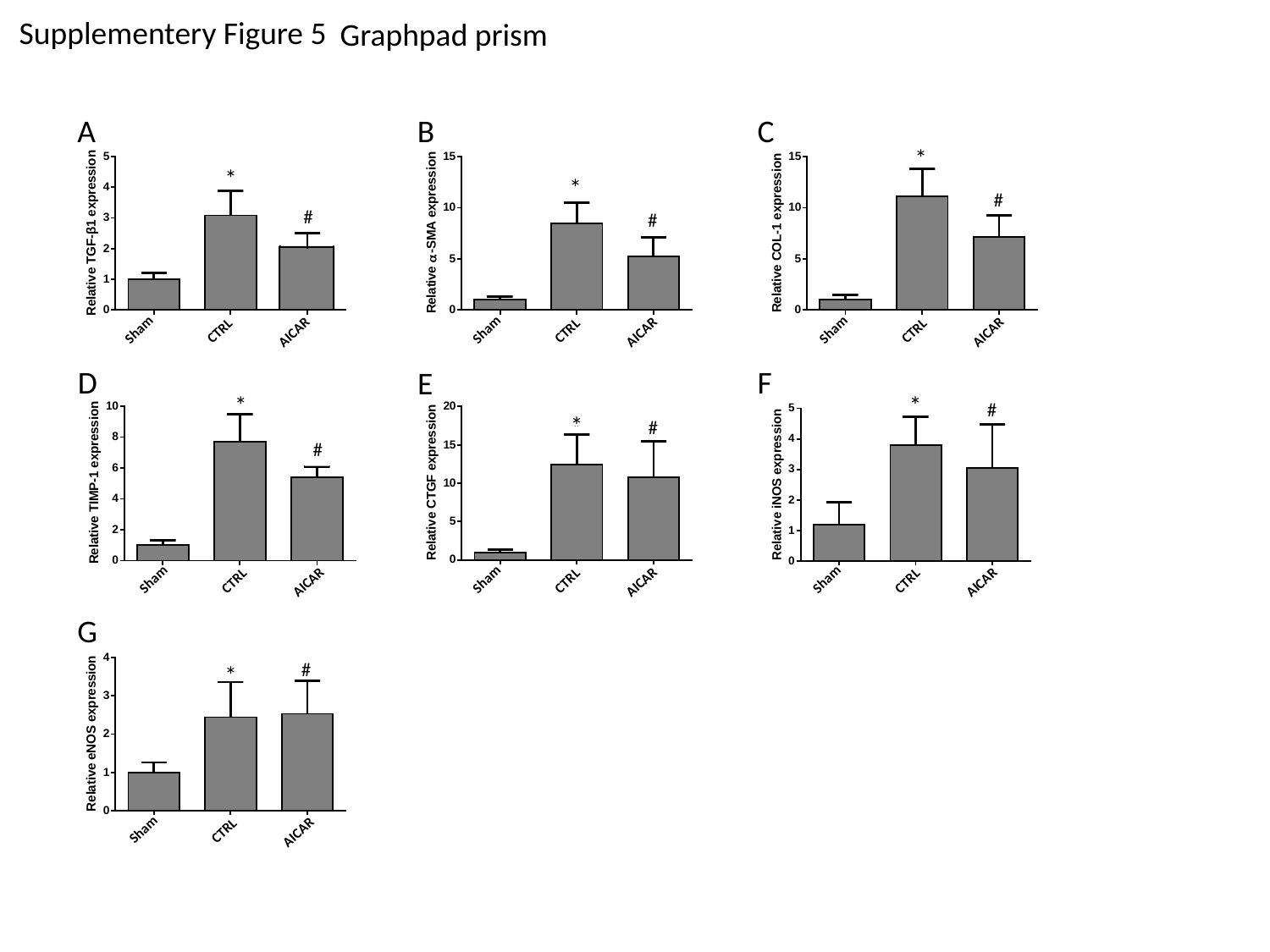

Supplementery Figure 5
Graphpad prism
A
C
B
 *
 *
 *
 #
 #
 #
CTRL
CTRL
CTRL
Sham
Sham
Sham
AICAR
AICAR
AICAR
 *
 *
 #
 *
 #
 #
CTRL
CTRL
CTRL
Sham
Sham
Sham
AICAR
AICAR
AICAR
 #
 *
CTRL
Sham
AICAR
D
F
E
G

## Slide 6
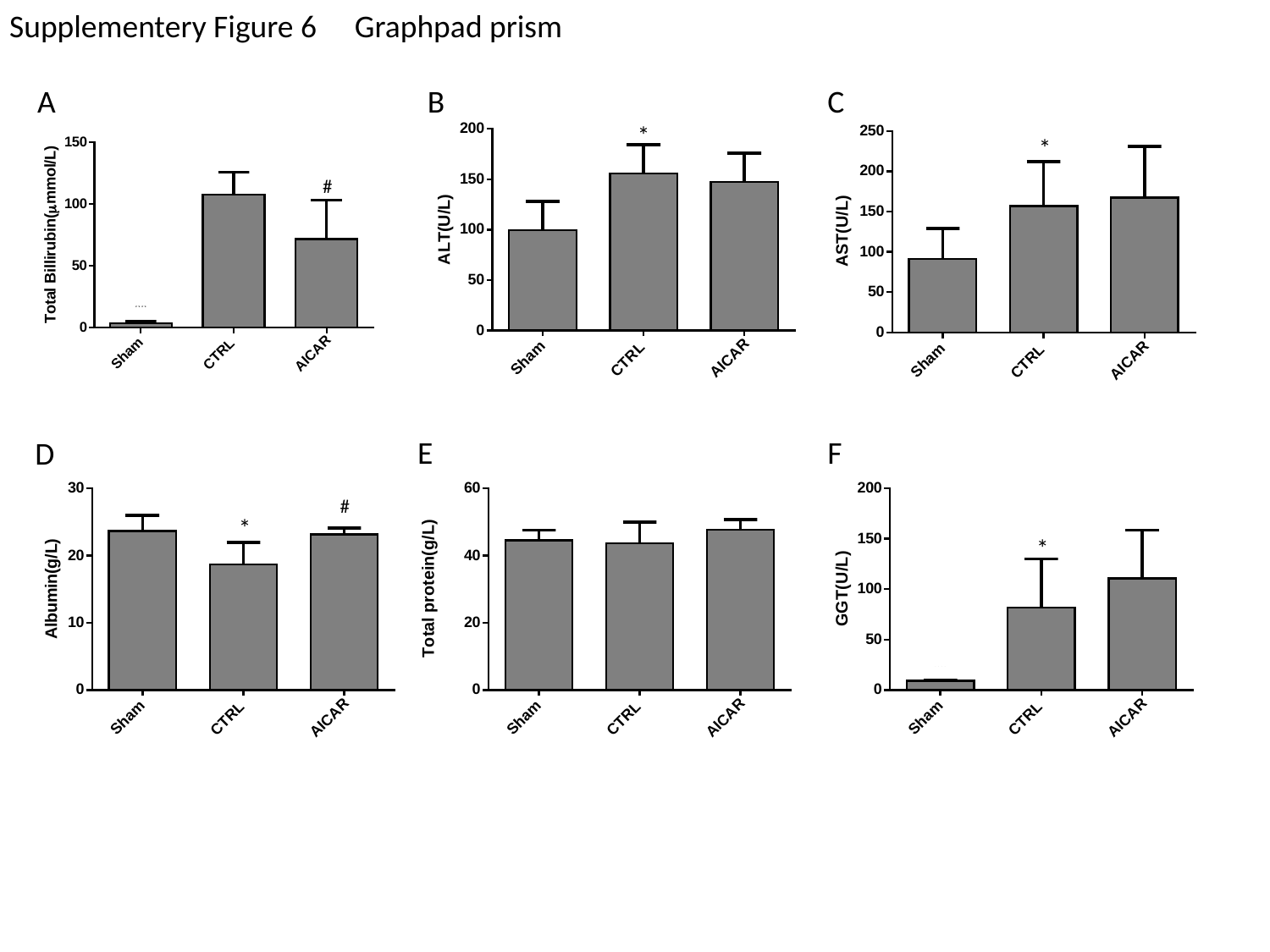

Graphpad prism
Supplementery Figure 6
C
A
B
 *
 *
 #
 #
 *
 *
F
E
D
